# New name for the soft coral *Alcyonium rubrum* Stokvis & van Ofwegen, 2006 (Alcyonacea, Alcyoniidae): *Alcyonium burmedju* nom. n.

**DOI:** 10.3897/zookeys.619.10086

**Published:** 2016-09-27

**Authors:** Íris Sampaio, Frank R. Stokvis, Leen P. van Ofwegen

**Affiliations:** 1 IMAR – Instituto do Mar & MARE – Marine and Environmental Sciences Centre, Universidade dos Açores, Departamento de Oceanografia e Pescas, Rua Prof. Dr. Frederico Machado 9901-862, Horta, Açores, Portugal; 2 Senckenberg am Meer, Abteilung Meeresforschung, Südstrand 40, 26382, Wilhelmshaven, Germany; 3 Naturalis Biodiversity Center, P.O. Box 9517, 2300 RA Leiden, The Netherlands

**Keywords:** Octocorallia, zoological nomenclature, homonymy, East Atlantic

**Figure 1.**
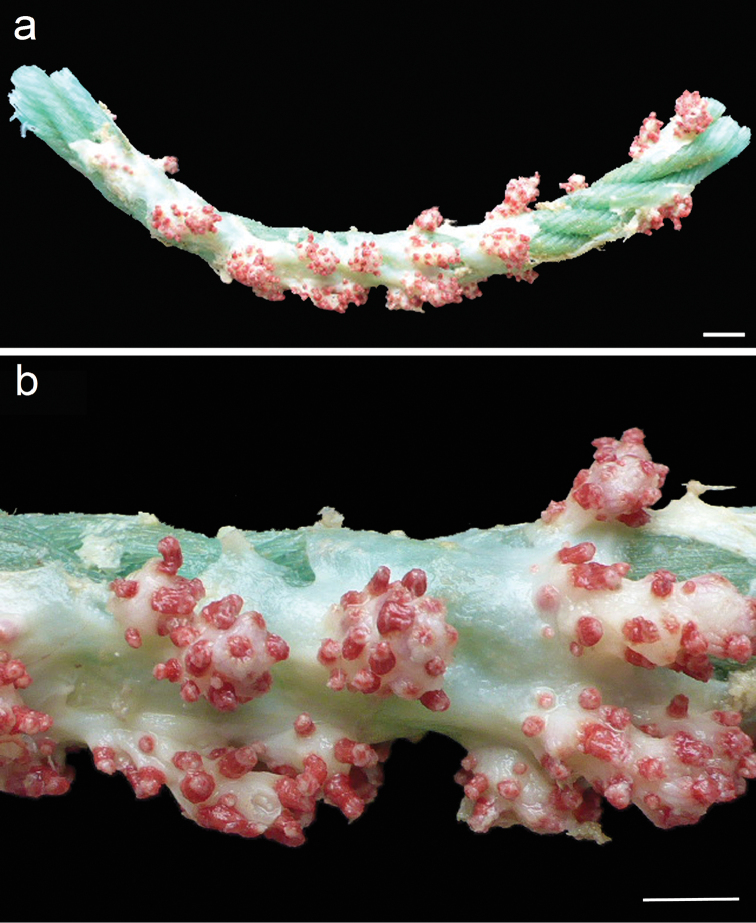
Holotype (RMNH Coel. 33879) of *Alcyonium rubrum* Stokvis & van Ofwegen, 2006, renamed *Alcyonium burmedju* nom. n. **a** Fishing rope serving as substrate for the encrusting soft coral (scale bar: 10 mm) **b** Detail of the colony. Scale bar: 5 mm.

*Alcyonium rubrum* Stokvis & van Ofwegen, 2006, an encrusting soft coral (Figure 1), was described from the Northeast Atlantic Ocean based on specimens collected during the Dutch CANCAP VII Expedition to the Cape Verde Archipelago (Stokvis and van Ofwegen 2006). This species was later reported from the Azores (Braga-Henriques et al. 2013).

A review on the taxonomic literature of octocorals by the first author revealed the existence of a species described from Scandinavia under the same name, *Alcyonium rubrum* Müller, 1776, which was also reported from Ireland (Hassal 1841). In such a case of primary homonomy, the International Code of Zoological Nomenclature Article 60, states that the junior homonym is invalid and needs to be replaced by a new name. We propose to replace *Alcyonium rubrum* Stokvis & van Ofwegen, 2006 by *Alcyonium burmedju* nom. n.

**Etymology.** The epithet “burmedju” means red in the criolo language spoken on Santiago Island in the Cape Verde Archipelago. The type locality is south of Raso islet, also localized in this island group (Stokvis and van Ofwegen 2006). Red is the predominant polyp colour of this species (Figure 1).
